# Preliminary assessment of an MRI-based grading system for leptomeningeal disease: an exploratory prognostic framework

**DOI:** 10.1007/s00234-026-03904-1

**Published:** 2026-01-26

**Authors:** Ahmed Msherghi, Maria Glue-Monroe, Rami W Eldaya, Maryam Pirhoushiaran, Heba Al Qudah, Ceylan Altintas Taslicay, Sahar Alizada, Hamza A. Salim, Leomar Y. Ballester, Max Wintermark

**Affiliations:** 1 Department of Neuroradiology, The University of Texas MD Anderson Cancer Center, Houston, TX USA; 2 Division of Pathology and Laboratory Medicine, The University of Texas MD Anderson Cancer Center, Houston, TX USA; 3 Department of Radiology, The University of Texas Southwestern Medical Center, Dallas, TX USA

**Keywords:** Leptomeningeal disease, Magnetic resonance imaging, Diagnostic criteria, Cancer metastasis, Neuro-oncology, Prognostic assessment

## Abstract

**Background:**

Current diagnostic approaches of leptomeningeal disease (LMD) rely heavily on cerebrospinal fluid (CSF) cytology, which shows significant limitations and the requirement for invasive procedures. We aim to develop an MRI-based grading scores for LMD diagnosis and prognosis that address current diagnostic limitations and provide standardized, reproducible assessment criteria.

**Methods:**

We conducted a retrospective analysis of 32 adult cancer patients evaluated for suspected LMD. Two experienced neuroradiologists independently assessed MRI studies using our novel grading system, which incorporates leptomeningeal enhancement/intensity patterns (grades 1–6), Evans index for hydrocephalus assessment, brain metastases characteristics, and spinal involvement. Confirmation of LMD cases was employed using dual confirmation approach combining CSF cytology and follow-up MRI.

**Results:**

Our MRI grading system demonstrated promising inter-observer performance. Inter-rater reliability between two attending level neuroradiologists was excellent (ICC = 0.953, P-value < 0.001) using a cutoff score of 2 or higher, the system demonstrated comparable performance. Risk stratification analysis revealed clear prognostic value, with mortality rates of 8.6% for low-risk patients (Grade 1–2), 50% for medium-risk patients (Grade 3–4), and 80.0% for high-risk patients (Grade 5 +). The Kaplan–Meier survival curves demonstrate a statistically significant difference in overall survival between patients with varying grades (p-value of 0.00011). Notably, survival probability drops steeply in the Grade 5 + group early on, suggesting that higher LMD burden is associated with rapid clinical deterioration. In contrast, low risk patients appear to have a more indolent course.

**Conclusions:**

Our preliminary findings detail a promising approach in evaluating LMD patients which offers valuable prognostic information for clinical decision making. Furthermore, the high inter-rater reliability across various tumor types further encourages the potential utility of this approach, although further research on a broader population is needed before clinical implementation.

**Supplementary Information:**

The online version contains supplementary material available at 10.1007/s00234-026-03904-1.

## Introduction

Neoplastic leptomeningeal disease (LMD), also known as leptomeningeal carcinomatosis, is a devastating complication of cancer in which malignant cells spread through the cerebrospinal fluid (CSF) and leptomeninges [1]. It occurs in 5–15% of patients with solid tumors, with higher rates in breast cancer (5–35%), lung cancer (10–26%), and melanoma (5–15%), translating to ~ 110,000 new U.S. cases annually [2–6].

Diagnosis of LMD remains difficult. Patients present with nonspecific symptoms such as headaches, confusion, seizures, or cranial neuropathies. The current “gold standard,” CSF cytology, has an average sensitivity of 50–60% on the first lumbar puncture, rising to 75–80% with repeated taps, and, negative cytology cannot reliably exclude LMD [7–10]. This poor sensitivity creates a frustrating clinical scenario where negative CSF cytology results cannot reliably rule out LMD, often requiring repeated spinal taps with the associated risks and patient discomfort.

The median survival of LMD patients limited to only 3–6 months despite available therapies [6, 11, 12]. By the time LMD is confirmed, disease is usually widespread, which restricts the impact of intrathecal therapy or focal radiation. Current diagnostic practice categorizes patients in a binary fashion either “LMD present” or “absent” without accounting for the extent of leptomeningeal involvement. This approach delays intervention and fails to stratify patients by risk. Importantly, there is no standardized tool to establish a reliable baseline measure of disease burden, which is essential both for prognosis and for monitoring treatment response over time. Our aim is to develop a standardized, semi-quantitative MRI-based grading system for LMD and move beyond binary diagnosis to capture disease extent and patterns in a reproducible way. This aims to primarily provide prognostic value and a framework for monitoring treatment response across tumor types and institutions and to assess inter-rater reliability of using such approach.

## Methods

### Study design and patients

This study was conducted in accordance with the Strengthening the Reporting of Observational Studies in Epidemiology (STROBE) guidelines [13]. We conducted a retrospective study at MD Anderson Cancer Center, with approval from the Institutional Review Board. Our cohort consisted of 32 adult cancer patients (aged 18 years or older) who were evaluated for suspected LMD between January 2022 and December 2023. Patients were followed up until June 30, 2025, to assess the overall survival.

**Fig. 1:**
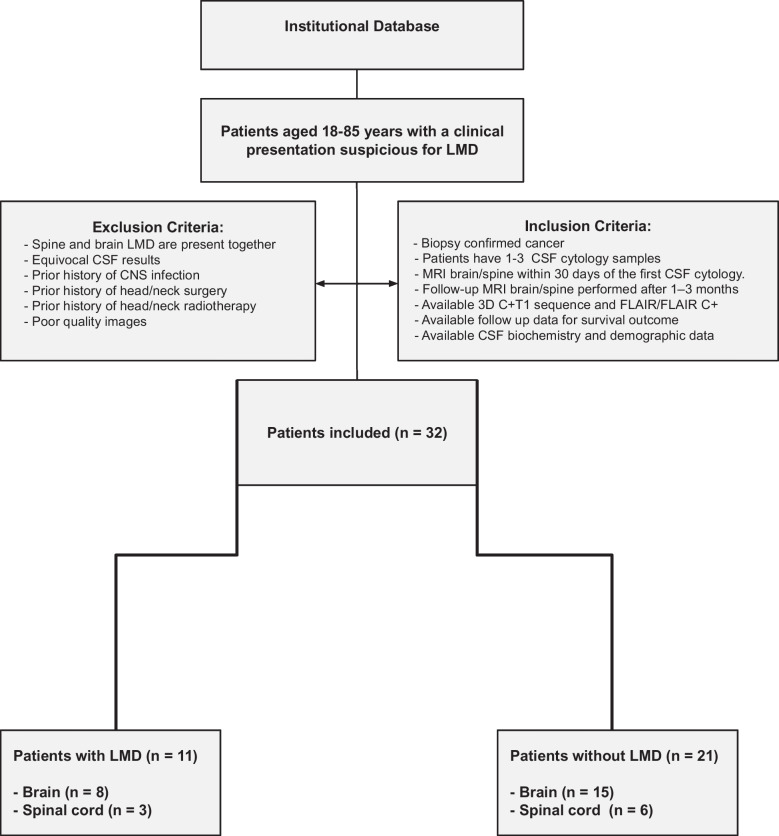
Inclusion and exclusion criteria of the study workflow

Patients were included if they had a biopsy-confirmed malignancy, clinical suspicion of LMD based on neurological symptoms (headache, altered mental status, cranial nerve deficits, seizures), a brain MRI with gadolinium contrast performed within 30 days of CSF analysis (cytology and chemistry), adequate image quality, and available follow-up imaging to confirm the diagnosis. Patients with concurrent CNS infections, recent neurosurgery, or head/neck radiotherapy, and those with both brain and spinal cord metastasis were not enrolled (Fig. 1).

### Imaging protocol

All MRI examinations utilized 3D 1.5 T or 3.0 T scanners with a standardized protocol that included three essential sequences: pre-contrast FLAIR, post-contrast FLAIR acquired 5–10 min after VUEWAY® (gadopiclenol) administration, and three-dimensional post-contrast T1-weighted sequences. Gadopiclenol is a newly introduced, high‑relaxivity, macrocyclic GBCA approved at a half-standard gadolinium dose and offering comparable lesion visualization and contrast enhancement to older agents, with a favorable safety profile and potential for reduced gadolinium exposure [14]. All MRIs were acquired using 3D post-contrast T1-weighted MPRAGE/VIBE-type sequences, depending on scanner platform. Importantly, no black-blood post-contrast sequences (such as T1-SPACE) were used in the diagnostic MRI studies included. This avoided the known pitfall where blood-suppression techniques can create linear or nodular enhancement/hyperintensity patterns that may mimic Grade 2 LMD on our scale.

### MRI grading system

#### Main grading

**Fig. 2:**
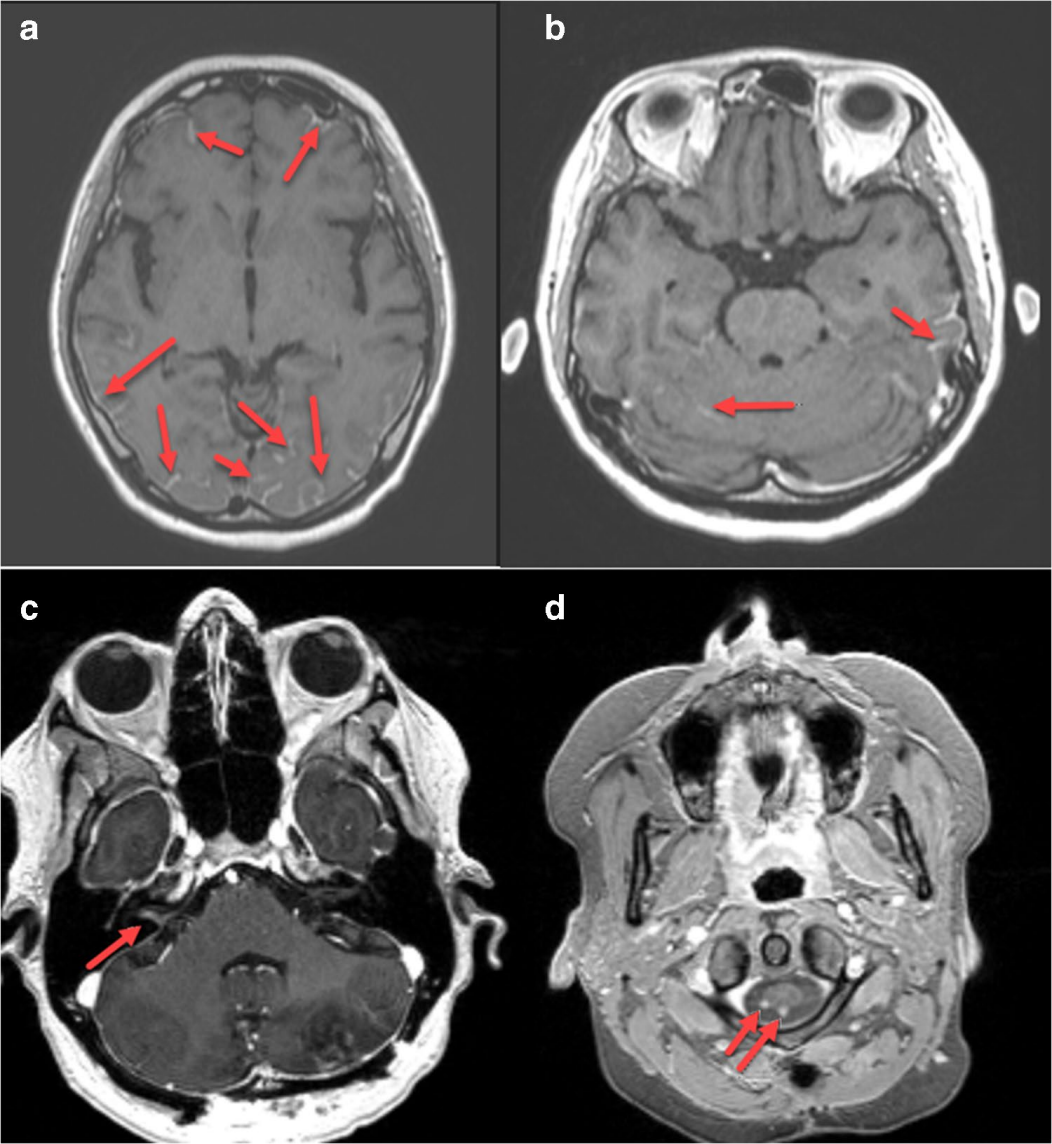
Examples of MRI imaging criteria in LMD patients. Representative examples of leptomeningeal disease (LMD) grades on post-contrast T1-weighted MRI. Panel (**a**) shows Grade 6 LMD, characterized by abnormal leptomeningeal enhancement/hyperintensity extending into the adjacent brain parenchyma, indicating invasive disease and the most aggressive form of LMD. Panel (**b**) demonstrates Grade 3 LMD, with diffuse thin (< 1 mm) linear enhancement/hyperintensity involving multiple sulci without nodularity, consistent with widespread but lower-burden leptomeningeal spread. Panel (**c**) also illustrates Grade 2 LMD, with focal thin enhancement/hyperintensity affecting auditory nerve. Panel (**d**) depicts Grade 5 LMD, showing diffuse, thick (> 1 mm) and nodular leptomeningeal enhancement/hyperintensity across multiple regions in the cervical spine, indicating a high tumor burden

Leptomeningeal Enhancement/hyperintensity Grades (1–6): We first defined six grades to categorize the extent and morphology of LMD on MRI (Fig. 2). Grades progress from Grade 1 (no evidence of LMD) through Grade 2 (equivocal focal thin enhancement/hyperintensity), Grade 3 (diffuse thin enhancement/hyperintensity), Grade 4 (focal thick/nodular enhancement/hyperintensity), Grade 5 (diffuse nodular thickening), to Grade 6 (parenchymal invasion with extensive nodular disease). At least grade 2 is required for considering additional scoring; those who scored 1 in the main scoring criteria were considered negative for LMD. In those who scored 2 or more in the main grading system, additional one point was assigned for Evans’ Index assessment of > 0.25 [15], and additional points for the presence of parenchymal metastatic masses (one point for single, and two points for multiple masses). A total score of 0–9 was retrieved. Please see the full details in Supplementary file 1.

#### Image analysis

Two experienced neuroradiologists (M.GM., with 10 years of experience post neuroradiology fellowship, and R.W.E., with 7 years of experience post neuroradiology fellowship) independently reviewed the studies while remaining completely blinded to clinical information, CSF results, and outcomes. Interrater agreement between their assessments was evaluated. For survival outcome analysis, a consensus reading was used, and any disagreements were resolved by a senior neuroradiologist (–., with 25 years of experience).

### Reference standard

Patients were classified as LMD-positive if they had positive CSF cytology (from up to three samples within a month) and MRI brain within one month from the date of the first CSF sample. Additionally, of disease persistence or progression on follow-up MRI at 1–3 months after the initial diagnosis. Patients were considered LMD-negative if they had negative CSF cytology or initial and subsequent negative follow-up imaging.

### Statistical analysis

Statistical analyses of descriptive data were conducted using SPSS (version 24). Categorical variables were summarized as frequencies and percentages, while continuous variables were described using medians, interquartile ranges (IQR), and full ranges. Associations between categorical variables were assessed using Fisher’s exact test and the Kruskal–Wallis test, given the non-parametric distribution of most variables in the study sample. Diagnostic accuracy analyses were performed using Python (version 3.11.8) with NumPy (1.26.4), SciPy (1.13.0), and Pandas (2.2.2). Inter-rater reliability was evaluated using intraclass correlation coefficients (ICC), derived from a two-way random-effects model for absolute agreement based on single measurements. Survival analysis was conducted employing Kaplan–Meier curves and log-rank testing to assess statistical significance. A two-sided significance level of α = 0.05 was applied throughout. All image measurements and segmentations were performed using 3D Slicer (version 5.6.2).

## Results

### Patient characteristics

Our final cohort included 32 patients, consisting of 11 LMD-positive and 21 LMD-negative subjects. Among the LMD-positive group, 8 patients had intracranial LMD and 3 had spinal LMD. Among the LMD-negative patients, 15 underwent brain MRI for evaluation, and 6 were assessed for suspected spinal LMD (Table 1). The median age was 63.5 years (range, 30–85), comprising 18 females (56.2%) and 14 males (43.8%). Primary cancers included breast cancer in 6 (18.8%) patients, lung cancer in 5 (15.6%) patients, melanoma in 2 (6.2%) patients, primary CNS tumors in 15 (46.9%) patients, and hematological malignancy in 4 (12.5%) patients. The brain was primarily studied in 23 (71.9%) patients, while 9 (28.1%) patients assessed for spine cord involvement. The most common presenting symptoms were headache in 25 (78.1%) patients, altered mental status in 18 (56.2%), peripheral nerve deficits in 14 (43.8%), and seizures in 10 (31.2%) patients. The median time from symptom onset to MRI was 14 days (range, 3–45 days), and the median interval between MRI and CSF analysis was 3 days (range, 0–28 days). Follow-up MRI was obtained at a median of 42 days (range, 28–84 days).

**Table 1:** Baseline characteristics and clinical outcomes comparing LMD-positive and LMD-negative patients

Characteristic	Total Cohort (N = 32)	LMD-Positive (n = 11)	LMD-Negative (n = 21)	P-value
Age, years Median (range)	63.5 (30–85)	65.0 (45–82)	62.0 (30–85)	0.58
Sex, *n* (%)				0.99
Male	14 (43.8)	5 (45.5)	9 (42.9)	
Female	18 (56.2)	6 (54.5)	12 (57.1)	
Location of suspected LMD, *n* (%)				0.99
Brain	23 (71.9)	8 (72.7)	15 (71.4)	
Spinal cord	9 (28.1)	3 (27.3)	6 (28.6)	
Primary Cancer Type, *n* (%)				0.47
Breast cancer	6 (18.8)	3 (27.3)	3 (14.3)	
Lung cancer	5 (15.6)	2 (18.2)	3 (14.3)	
Melanoma	2 (6.2)	1 (9.1)	1 (4.8)	
Primary CNS tumors	15 (46.9)	4 (36.4)	11 (52.4)	
Other tumors	4 (12.5)	1 (9.1)	3 (14.3)	
Clinical Presentation, *n* (%)				
Headache	25 (78.1)	10 (90.9)	15 (71.4)	0.37
Altered mental status	18 (56.3)	8 (72.7)	10 (47.6)	0.26
Peripheral nerve deficits	14 (43.8)	7 (63.6)	7 (33.3)	0.14
Seizures	10 (31.3)	5 (45.5)	5 (23.8)	0.25
Other symptoms	12 (37.5)	6 (54.5)	6 (28.6)	0.25
CSF Cytology, *n* (%)				0.002*
Positive	13 (40.6)	9 (81.8)	4 (19.0)	
Negative	19 (59.4)	2 (18.2)	17 (81.0)	
CSF Biochemistry				
Elevated protein (> 45 mg/dL), *n* (%)	26 (81.3)	10 (90.9)	16 (76.2)	0.63
Decreased glucose (< 60 mg/dL), *n* (%)	18 (56.3)	8 (72.7)	10 (47.6)	0.26
Elevated WBC (> 5 cells/μL), *n* (%)	22 (68.8)	9 (81.8)	13 (61.9)	0.42
Imaging Characteristics				
Time from symptoms to MRI, days (median, range)	14 (3–45)	12 (5–35)	15 (3–45)	0.654
Time between MRI and CSF, days (median, range)	3 (0–28)	2 (0–21)	4 (0–28)	0.423
Follow-up MRI, days (median, range)	42 (28–84)	38 (28–65)	44 (28–84)	0.512
MRI Grading System Results				< 0.001*
MRI score, median (range)	2 (1–8)	5 (3–8)	1 (1–2)	
Risk Stratification, *n* (%)				< 0.001*
Low risk (score ≤ 2)	23 (71.9)	2 (18.2)	21 (100)	
Medium risk (score 3–4)	4 (12.5)	4 (36.3)	0	
High risk (score ≥ 5)	5 (15.6)	5 (45.5)	0	
Mortality	**Low Risk**	**Medium Risk**	**High Risk**	**0.005**
Alive	21 (91.3)	2 (50)	1 (20)	
Died	2 (8.6)	2 (50)	4 (80)	

Moreover, elevated CSF protein (> 45 mg/dL) was observed in 26 of 32 patients (81.3%), including 10 of 11 (90.9%) in the LMD-positive group and 16 of 21 (76.2%) in the LMD-negative group. Decreased glucose levels (< 60 mg/dL) were present in 18 patients (56.3%), occurring in 8 of 11 (72.7%) LMD-positive patients and 10 of 21 (47.6%) LMD-negative patients. Elevated CSF white blood cell count (> 5 cells/μL) was noted in 22 patients (68.8%), including 9 of 11 (81.8%) LMD-positive patients and 13 of 21 (61.9%) LMD-negative patients. Although these biochemical abnormalities were more frequent in the LMD-positive group across all parameters, the differences did not reach statistical significance. (Table 1).

### Detection rate

**Fig. 3:**
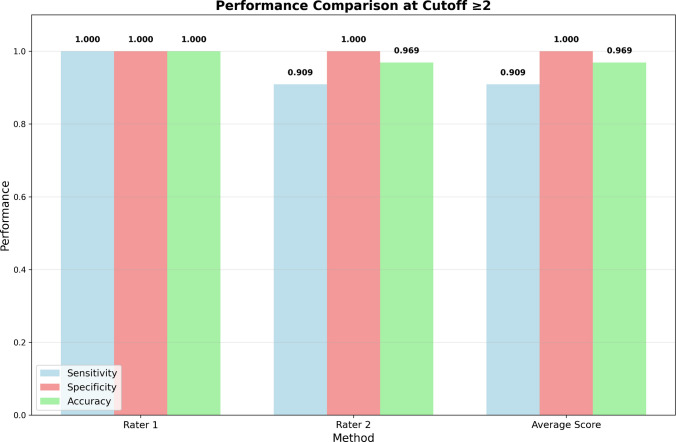
Diagnostic performance of individual raters and combined assessment

**Fig. 4:**
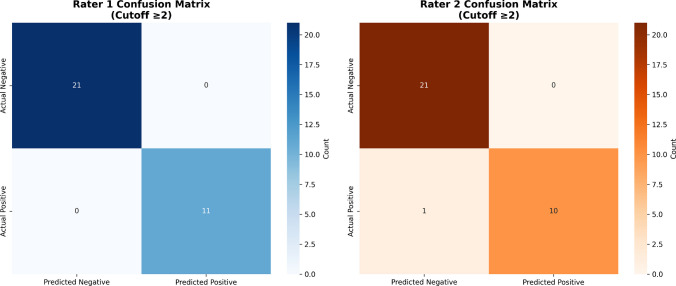
Confusion matrices depicting individual rater evaluations. The single discordant case represented a false-positive classification and involved thin, focal leptomeningeal enhancement that met criteria for Grade 2 under our proposed system but did not meet the diagnostic standard based on CSF cytology or follow-up MRI. On retrospective review, the imaging appearance was favored to represent a vascular structure rather than true LMD, underscoring the inherent difficulty of interpreting very subtle findings

LMD-positive patients had significantly higher MRI scores (median 5 points, range 3–8) compared to LMD-negative patients (median 1 point, range 1–2 *p* < 0.001). Choosing a cutoff of 2 points or more keeping in mind the obligatory condition of at least scoring one point from the main LMD grading, the scale detected the majority of cases with the overall diagnostic accuracy of 96.9% (95% CI: 81.0–99.5%). See Figs. 3 and 4.

### Inter-rater reliability

**Fig. 5:**
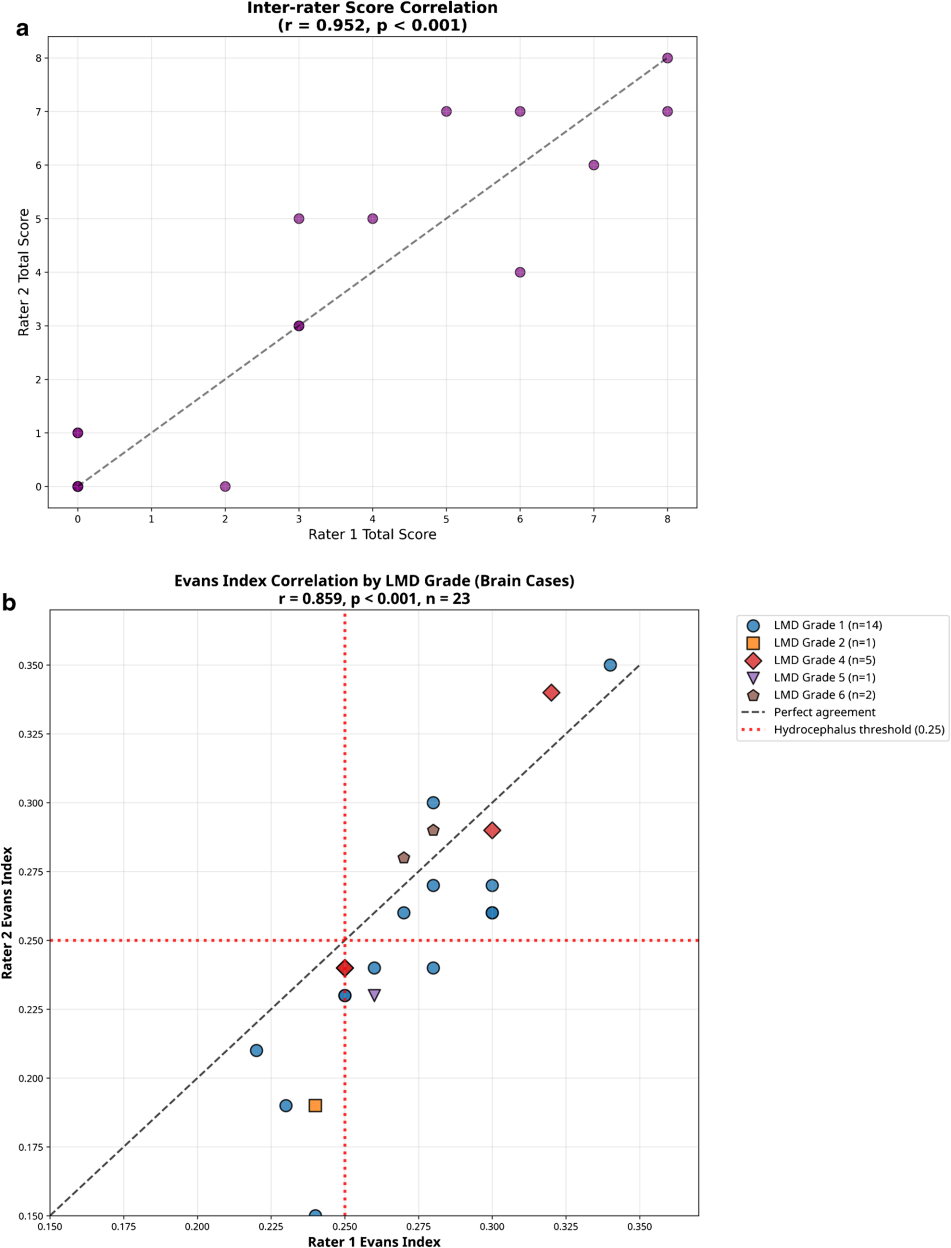
**a** Inter-rater agreement for MRI-based tumor burden scoring. **b** Inter-rater reliability of evans index measurements on MRI

The agreement between the two independent readers was excellent, with an overall ICC of 0.952 (95% CI: 0.883–0.977) for the total MRI scores, indicating near-perfect reproducibility as shown in Fig. 5a, b. This high level of consistency suggests that the grading system is both reliable and robust across observers, reinforcing its potential clinical utility. The strong inter-rater reliability also supports the applicability of the proposed MRI score in routine clinical practice and multi-center research settings, where standardized interpretation is essential.

### Prognostic value

**Fig. 6:**
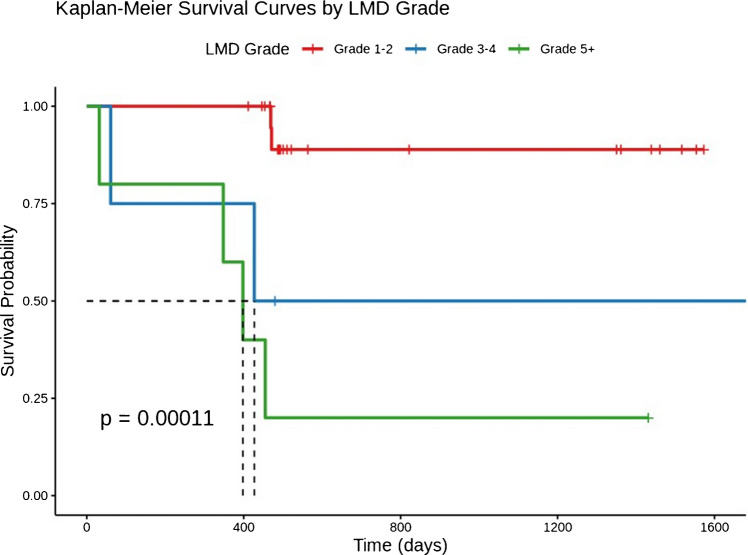
Kaplan–Meier survival curves stratified by study risk groups

Patients were assessed for overall all-cause mortality following the imaging evaluation. Patients were then stratified into three risk groups: low-risk (score ≤ 2, *n* = 23), medium-risk (score 3–4, *n* = 4), and high-risk (score ≥ 5, *n* = 5). During follow-up through June 2025, mortality rates were 9.1% for low-risk patients, 50% for medium-risk patients, and 80.0% for high-risk patients (*P* = 0.005). Additionally, Kaplan–Meier survival analysis revealed a statistically significant difference in overall survival among patients stratified by LMD risk category (log-rank *p* = 0.00011) (Fig. 6).

## Discussion

Our MRI grading system introduces a clear, six-tiered framework focused mainly on the extent and morphology of leptomeningeal enhancement/hyperintensity from absent (Grade 1) to aggressive parenchymal invasion (Grade 6), and supplementary scoring for clinically relevant features like hydrocephalus and intracranial metastases. In contrast, the original RANO/LANO scorecard from 2019 encompasses a broader range of features, including cranial nerve enhancement/hyperintensity, spinal nodules, and measurable lesion change quantified on a − 3 to + 3 scale. However, field validation found the LANO scorecard faced substantial implementation challenges with significant inter-rater variability,. For single-item assessment at baseline, Krippendorff’s alpha coefficients were consistently low ranging from 0.40 to 0.43 for brain-related items such as nodules and leptomeningeal enhancement/hyperintensity, and only marginally better (0.45–0.60) for spinal features like nodules and nerve root enhancement/hyperintensity [16]. By concentrating on LM-specific imaging findings and employing a streamlined scoring structure, our system aims to enhance inter-rater consistency, clinical relevance, and applicability thereby paving the way for more reliable LM response assessment.

The detection rate of patients using our model reached 96.9%, which is comparable to the published rates for CSF cytology, which range from 65 to 95% [16–21], and Conventional MRI interpretation that ranges between 70 and 90% [1, 5–8, 22]. Moreover, the inter-rater reliability of our independent imaging experts substantially exceeds reported values for subjective imaging assessment [23]. These improvements could have positive implications for both the quality of clinical care and the standardization of research.

Furthermore, our report presents preliminary data supporting the development of a novel prognostic methodology that is lacking in current approaches. The clear separation into risk groups, with different mortality rates (8.6%, 50%, and 80%), points toward clinically actionable information for treatment planning and patient counseling. This prognostic capability could help guide treatment intensity decisions and resource allocation. The strong correlations between imaging findings and outcomes further support the potential usability of our approach. Leptomeningeal enhancement/hyperintensity reflects the underlying disease burden, with more extensive involvement corresponding to a greater disease burden and a worse prognosis. Moreover, the inclusion of semi-quantitative measures, such as the Evans index, provides additional information about disease complications that affect clinical management. The consistent performance across different cancer types, such as breast, lung, melanoma, and primary CNS tumors, demonstrates that leptomeningeal enhancement/hyperintensity patterns are determined by anatomical and physiological factors rather than primary tumor characteristics, which potentially supports universal applicability and simplifies implementation across diverse clinical settings [6, 11].

Our system is compatible with routine clinical imaging protocols, utilizing widely available MRI sequences, and provides minimal barriers to implementation. The preliminary semi-quantitative and noninvasive approach may reduce dependence on subjective interpretation while providing clear guidance for clinical decisions. It could be integrated into modern radiology information systems and potentially automated using artificial intelligence approaches. Additionally, the selected population in our pilot analysis exhibits a short temporal correlation between Imaging and CSF analysis (median 3 days), ensuring that our comparisons reflect clinically relevant scenarios where both assessments are typically available for clinical decision-making. However, further validation of external and larger datasets is needed to support our findings.

## Limitations

Several limitations should be acknowledged. Our retrospective, single-institution design with a cohort of 32 patients limits the generalizability of our findings, and therefore, the prognostic findings should be regarded as hypothesis-generating rather than a confirmatory report as it is unadjusted, for confounding factors due to the smaller sample size. Larger, prospective, multi-institutional studies are warranted to further stratify patients based on clinical characteristics, ensure proper matching of cases and controls, and perform adjusted survival analyses, such as Cox proportional hazards modeling using larger datasets. The requirement for gadopiclenol contrast may also limit applicability in patients with contraindications such as prior hypersensitivity reactions, pregnancy, or renal impairment. Nevertheless, these pilot findings lay the groundwork for future prospective validation studies, which are essential for clinical implementation. This study included MRI examinations acquired on both 1.5 T and 3 T scanners, which may suggest broader applicability of the proposed grading system. However, given the relatively small sample size, we were unable to adequately assess scanner-related variability or the robustness of the grading system across different field strengths. This should therefore be interpreted as a limitation of the current study. Future research should focus on single-etiology leptomeningeal disease cohorts and leverage multi-institutional collaborations to produce more robust and generalizable results that clinicians can confidently apply in practice.

## Supplementary Information

Below is the link to the electronic supplementary material.

Supplementary file1 (DOCX 21 KB)

## Data Availability

De-identified data supporting the conclusions of this article are available from the corresponding author upon reasonable request and with appropriate institutional approvals.

## Declarations

### Use of AI tools

Generative artificial intelligence tools were not involved in the study's design, data analysis, interpretation, or manuscript preparation. Their use was limited exclusively to minor language refinement to enhance readability, without contributing to the generation of scientific content or influencing the study's methodology or conclusions.
