# Supplementary material for: Preliminary assessment of an MRI-based grading system for leptomeningeal disease: an exploratory prognostic framework
Source: Neuroradiology. 2026 Jan 26;68(6):1651–60. doi: 10.1007/s00234-026-03904-1 (PMC13323362; doi:10.1007/s00234-026-03904-1)
Supplement: Supplementary file 1 — Supplementary file1 (DOCX 21 KB) [file 234_2026_3904_MOESM1_ESM.docx]

*This supporting document is part of the following manuscript****:*** Preliminary Assessment of an MRI-Based Grading System for Leptomeningeal Disease: An Exploratory Prognostic Framework

**MRI Grading Score**

***Main Grading***

Leptomeningeal enhancement/hyperintensity Grades (1–6): We defined six grades to categorize the extent and morphology of leptomeningeal disease on MRI **(Figure 1)**:

Grade 1: No evidence of LMD. There is no abnormal leptomeningeal enhancement/hyperintensity or FLAIR hyperintensity suggestive of LMD. The meninges appear normal. (This corresponds to an LMD-negative scan.)

Grade 2: Focal thin Enhancement/hyperintensity. A localized area of linear, thin equivocal leptomeningeal enhancement/hyperintensity is present, <1 mm in thickness, typically along the pial surface. This could appear as an isolated linear enhancement/hyperintensity along a cortical sulcus or cranial nerve sleeve. Only one region is involved (focal), and the enhancement/hyperintensity is subtle and thin.

Grade 3: Diffuse thin enhancement/hyperintensity. Thin linear enhancement/hyperintensity involving two or more regions or widely distributed. The leptomeningeal enhancement/hyperintensity is still <1 mm in thickness everywhere (no nodularity), but it is diffuse, meaning it affects multiple sulci or cisterns across the brain (e.g., coating the cerebral convexities in multiple lobes). This pattern suggests a more extensive spread, but without frank thickening.

Grade 4: Focal thick enhancement/hyperintensity. A localized area of nodular or thick leptomeningeal enhancement/hyperintensity is seen, defined as >1 mm in maximal thickness at that site. This usually appears as a focal nodular deposit or plaque of enhancement/hyperintensity on the meninges (for example, a thick clump of enhancement/hyperintensity in the basal cistern or along the spinal cauda equina on brain or spine imaging). Only one or two regions show this nodular enhancement/hyperintensity (not widespread).

Grade 5: Diffuse thick enhancement/hyperintensity. There are two or more areas of nodular thickening (>1 mm) or a general thickening of the meninges visible throughout various regions. In other words, nodular deposits are present in a diffuse distribution. This is a severe pattern, indicating heavy leptomeningeal tumor burden (e.g., numerous enhancing nodules along the cerebellar folia, cerebral sulci, and ventricular linings).

Grade 6: Leptomeningeal enhancement/hyperintensity with parenchymal invasion. This highest grade is assigned when leptomeningeal disease invades the adjacent brain parenchyma, blurring the distinction between meningeal and parenchymal tumors. For example, one might observe tumor extension from the surface into the cortical ribbon or along perivascular spaces into the brain, or tumoral nodules breaking through into the brain tissue. This represents the most aggressive manifestation of Imaging and often coexists with extensive nodular disease (thus usually also diffuse).

***Additional points***

In preliminary analysis, we identified three other MRI features that are clinically relevant and correlate with the presence and severity of LMD. These are incorporated as additional points in the scoring:

Presence of ventriculomegaly disproportionate to age ^1^, quantified by an Evans' index greater than 0.25 (the ratio of frontal horn width to inner skull diameter). Dilated ventricles in a cancer patient with possible brain insult can result from blockage of CSF flow by leptomeningeal tumor deposits in the ventricles or basal cisterns. If Evans' Index is greater than 0.25, add 1 point ^2^.

Presence of Parenchymal metastases: Widespread metastatic disease in the brain is a risk factor for developing LMD because it indicates hematogenous spread and a high tumor load ^3^. If the MRI shows single distinct metastases or two or more, we assign 1 and 2 points, respectively. Two or more masses are slightly heavier than a single large mass because numerous smaller metastases increase the chance of at least one reaching the CSF.

1. Zhou X, Xia J. Application of Evans Index in Normal Pressure Hydrocephalus Patients: A Mini Review. *Front Aging Neurosci* 2021;13:783092

2. Le Rhun E, Devos P, Seystahl K, et al. Prognostic Role of Ventricular Size and Its Dynamics in Patients With Leptomeningeal Metastasis From Solid Tumors. *Neurology* 2024;102:e207959

3. Nguyen A, Nguyen A, Dada OT, et al. Leptomeningeal Metastasis: A Review of the Pathophysiology, Diagnostic Methodology, and Therapeutic Landscape. *Curr Oncol* 2023;30:5906-5931
